# Sal B targets TAZ to facilitate osteogenesis and reduce adipogenesis through MEK‐ERK pathway

**DOI:** 10.1111/jcmm.14272

**Published:** 2019-03-25

**Authors:** Na Wang, Yukun Li, Ziyi Li, Chang Liu, Peng Xue

**Affiliations:** ^1^ Department of Endocrinology Hebei Medical University Third Affiliated Hospital Shijiazhuang PR China; ^2^ Key Orthopaedic Biomechanics Laboratory of Hebei Province Shijiazhuang PR China

**Keywords:** adipogenesis, MEK‐ERK pathway, osteogenesis, Salvianolic acid B, TAZ

## Abstract

Salvianolic acid B (Sal B), a major bioactive component of Chinese herb, was identified as a mediator for bone metabolism recently. The aim of this study is to investigate the underlying mechanisms by which Sal B regulates osteogenesis and adipogenesis. We used MC3T3‐E1 and 3T3‐L1 as the study model to explore the changes of cell differentiation induced by Sal B. The results indicated that Sal B at different concentrations had no obvious toxicity effects on cell proliferation during differentiation. Furthermore, Sal B facilitated osteogenesis but inhibited adipogenesis by increasing the expression of transcriptional co‐activator with PDZ‐binding motif (TAZ). Accordingly, TAZ knock‐down offset the effects of Sal B on cell differentiation into osteoblasts or adipocytes. Notably, the Sal B induced up‐expression of TAZ was blocked by U0126 (the MEK‐ERK inhibitor), rather than LY294002 (the PI3K‐Akt inhibitor). Moreover, Sal B increased the p‐ERK/ERK ratio to regulate the TAZ expression as well as the cell differentiation. In summary, this study suggests for the first time that Sal B targets TAZ to facilitate osteogenesis and reduce adipogenesis by activating MEK‐ERK signalling pathway, which provides evidence for Sal B to be used as a potential therapeutic agent for the management of bone diseases.

**Hightlight**

1. Sal B had no obvious toxicity effects on cell proliferation.
2. Sal B increased TAZ to facilitate osteogenesis and inhibit adipogenesis.
3. Sal B activated MEK‐ERK pathway to regulate the TAZ expression and cell differentiation.

## 1 INTRODUCTION

Bone tissue rich in blood vessels is subjected to continuous remodeling.^1^, ^2^ However, it often fails when the healing capacity is compromised in many clinical situations such as osteoporosis and diabetes.^3^ Patients suffering from osteoporosis showed an increased number of adipocytes in their bone marrow with a reduction in the pool of mesenchymal stem cells (MSCs) differentiating into osteoblasts.^4^ In many cases, inducers of differentiation for one lineage often repress the cell differentiation into other types.^5^ This relationship is also observed between osteogenesis and adipogenesis.^6^, ^7^ These medical breakthrough stirs incredible interest in anabolic therapies for osteoporosis, whereby osteogenic differentiation is stimulated by preventing adipogenic differentiation simultaneously. Coincidentally, our study aims to explore the exact role of Sal B in osteogenesis and adipogenesis.

Salvia miltiorrhiza is a traditional Chinese medicine, called danshen and widely used in clinical practice for cardio‐cerebral vascular diseases.^8^, ^9^ Sal B, the major bioactive component of Salvia miltiorrhiza, is the most active constituent of water‐soluble salvianolic acid substances.^10^ The structure of Sal B is shown in Figure S1 which consists of three molecules of Tanshinol and one molecule of caffeic acid. Its molecular formula is C36H30O16. Studies have shown that Sal B exerted neuro‐protective effects and could alleviate liver fibrosis.^10^, ^11^ Recently, increasing attentions have been directed to the effects of Sal B on bone metabolism.^13^, ^14^ He et al^15^ reported that Sal B promoted bone formation by increasing activity of alkaline phosphatase (ALP) in a rat tibia fracture model. However, the underlying mechanisms by which Sal B improves bone remodeling have not been well established.

TAZ, a transcriptional modulator, is one of such regulators that have key roles in cell proliferation, differentiation and function.^16^, ^17^ As reported, TAZ could interact with kinds of transcription factors to activate or repress specific gene expression, which might influence cell functions.^19^, ^20^ Byun et al^19^ have discovered that the Phorbaketal A increased the TAZ expression to promote osteogenic differentiation as well as inhibit adipogenic differentiation. Our team has also reported several researches focusing on TAZ induced osteogenic differentiation.^21^ Thus, it attracted our interest that whether TAZ plays an important role in the Sal B‐regulated cell differentiation.

In addition to the changes of the endogenous factors which are controlling the cell lineage‐specific differentiation, this study indicated that Sal B administration altered the differential balance between osteogenesis and adipogenesis. Note that Sal B increased TAZ expression and facilitated the osteoblastogenesis at the expense of the reduced adipogenesis. We also pointed to a close link of MEK‐ERK signalling transduction to the TAZ transcriptional networks during osteoblasts and adipocytes maturation. Taken together, this study further provides evidence for Sal B to be used as a potential therapeutic agent for the management of bone diseases clinically.

## 2 MATERIALS AND METHODS

### 2.1 Cell culture and differentiation

MC3T3‐E1 cells were obtained from American Type Culture Collection (ATCC CRL‐2594) and cultured in alpha minimal essential medium (α‐MEM; Gibco, USA) supplemented with 12% fetal bovine serum (FBS; Gibco, USA) and 1% penicillin–streptomycin (Gibco, USA) in a humidified incubator at 37°C with 5% CO_2_. The culture media was refreshed every 2‐3 days. When the cells reached approximately 80% confluence, they were sub‐cultured into a new culture flask at a 1:2 ratio or the complete medium was replaced with osteogenic medium containing α‐MEM, 12% FBS, 1% penicillin–streptomycin, 10mM β‐glycerophosphoric acid (Sigma‐Aldrich) and 50 μg/mL ascorbic acid (Sigma‐Aldrich)].

3T3‐L1 cells were also obtained from American Type Culture Collection (ATCC CRL‐2594) and cultured in Dulbecco's modified Eagle's medium (DMEM‐LG; Gibco, USA) supplemented with 12% FBS, 1% penicillin‐streptomycin. Adipogenic differentiation was induced by culturing cells in the adipogenic cocktail [DMEM‐LG supplemented with 12% FBS, 1% penicillin‐streptomycin (Gibco, USA), 10mg/ml insulin (Sigma‐Aldrich Corp.), 500 mmol/L methyl‐isobutylxanthine (Sigma‐Aldrich Corp.) and 1 μmol/L dexamethasone (Sigma‐Aldrich Corp.)].

### 2.2 Sal B administration

When the cells reached approximately 80% confluence, they were sub‐cultured into a new culture flask and the growth medium were replaced by differential medium in the presence or absence of Sal B (0.1 mmol/L, 1 mmol/L, or 10 mmol/L). Then, we selected the best acting concentration of Sal B for subsequent experiments based on the expression of differential markers analysed by the real‐time reverse transcription‐polymerase chain reaction (real‐time RT‐PCR) analysis, Western blotting analysis and staining after the induction of the differential medium, considering the toxicity effects of Sal B at different concentration on cell proliferation.

### 2.3 Cell viability assay

Cells were seeded at a density of 5 × 10^4^ cells/100 μL/well in 96‐well plates and incubated for cell viability assay. After 3 days’ treatment, 20 μL of freshly prepared MTT (5 mg/mL; Solarbio) was added and the plates were incubated at 37°C for another 4 hours to form crystals. Then, 150 μL of dimethyl sulfoxide (DMSO) was added for 10 minutes to fully dissolve the crystals. Finally, the absorbance of each well was measured at a wavelength of 490 nm using the microplate spectrophotometer (BioTek Instruments, San Jose, CA).

### 2.4 Cell cycle analysis

To identify the effect of Sal B on cell cycles, 3 days treated cells were harvested and fixed with 70% ethanol. After washed with PBS, cells were stained with propidium iodide (Sigma, USA) (5 mg/mL) for 30 minutes in the dark at 4°C. Fluorescence was measured with the flow cytometer equipped with a 570 nm argon ion laser (Epics XL, Beckman Coulter Corporation, FL) and the data were analysed using the Muticycle AV software.

### 2.5 Plasmids transfection

The plasmid containing small interfering RNA (SiRNA) sequences against TAZ (SiTAZ) expression were designed and synthesized by Shanghai Genechem Corporation (Shanghai, China). Relatively vacant plasmids with same antibiotic resistance were used as the negative control for SiTAZ group, with a name of CON36. Cultured cells were transfected with Lipofectamine 3000 (Thermo Fisher Scientific, Waltham, MA) after reaching approximately 80% confluence, in accordance with the manufacturer's instructions. After being transfected for 24 hours, the cells were then switched to differential medium for osteogenesis or adipogenesis. Transfected cells expressing green fluorescent protein (GFP) reporter were observed under a fluorescence microscope (Leica, Wetzlar, Germany). And the transfection efficiency of each plasmid was measured by the flow cytometry (Epics XL, Beckman Coulter Corporation, FL) 3 days post‐transfection. Furthermore, the expression of TAZ in different groups was assessed using real‐time RT‐PCR and Western blotting analysis. The sequences of TAZ‐SiRNA were 5′‐GATCCCCTGGACCAAGTATATGAACCACTCGAGTGGTTCATATACTTGGTCCAGTTTTTGGAT‐3′; 5′‐AGCTATCCAAAAACTGGACCAAGTATATGAACCACTCGAGTGGTTCATATACTTGGTCCAGGG‐3′.

### 2.6 Alizarin Red staining

Cells were seeded in 35 mm plastic dishes (Costar) for Alizarin Red staining (AR‐S) after 14 days’ induction of the osteogenic cocktail. Cells were washed twice with PBS and fixed with 4% paraformaldehyde (Solarbio, China) at room temperature for 15 minutes. Then the dishes were washed three times with distilled water and incubated with 0.1% AR (Sigma, USA) at 37°C for 30 minutes. Cells were then washed thoroughly with distilled water three times and the images were acquired using the microscope (Leica, Wetzlar, Germany). For quantification, Alizarin Red was de‐stained with 10% cetylpyridinium chloride (Sigma, USA) for 30 minutes at room temperature. The calcium concentrations were determined by detecting the OD value at 562 nm wavelength with a microplate spectrophotometer (Leica, Wetzlar, Germany). All data were normalized to the total protein content.

### 2.7 Oil red O staining

Adipogenesis was verified by oil red o staining following the standard procedures after 14 days’ induction of adipogenic medium. The cells were cultured in 35 mm plastic dishes (Costar) dishes, washed with PBS and fixed with 10% formaldehyde for 15 minutes at room temperature. Oil red o stock solution [0.5 g powder (Sigma) in 100 ml isopropanol] was mixed 3:2 with deionized water and left at room temperature for 10 minutes before filtering. The filtered oil red o mix was added to the dishes for 30 minutes at room temperature. Cells were washed thoroughly with distilled water for at least three times to acquire the images of the staining cells by the microscope (Leica, Wetzlar, Germany). For quantification, the stained cells were de‐stained with isopropanol in PBS for 30 minutes at room temperature. Light absorbance by the extracted dye was measured at 520 nm using the microscope (Leica, Wetzlar, Germany).

### 2.8 Real‐time reverse transcription‐polymerase chain reaction

Total RNA was extracted using TRIzol^®^ reagent (Ambition). Total RNA (1 μg) was reversed‐transcribed into cDNA using RevertAid™ First Strand cDNA synthesis Kit (Thermo, Waltham, USA) following the manufacturer's recommendations. Real‐time reverse transcription‐polymerase chain reaction (Real‐time RT‐PCR) was performed on a CFX96 Real‐Time PCR Detection System (Bio‐Rad, Hercules, CA) using SuperReal PreMix Plus (TIANGEN, Beijing, China) according to the manufacturer's protocols. It was performed in a volume of 20 μL that included 2 μL of undiluted cDNA, 10 μL of 2 × SuperReal PreMix Plus, 0.6 μL of forward primer, 0.6 μL of reverse primer and 6.8 μL of Nuclease‐Free Water as follows: pre‐denaturation at 95°C for 15 minutes followed by 40 cycles of three steps (95°C for 10 seconds, 62°C for 20 seconds, and 72°C for 30 seconds). Each RNA sample was performed in duplicate. GAPDH was used as housekeeping genes for normalizing mRNA levels. All primers were synthesized by Invitrogen (Carlsbad, CA). The relative expression of mRNAs were calculated according to the ratio of the copy numbers of the target genes [TAZ, runt related transcription factor 2 (RUNX2), osteocalcin (OCN), CCAAT/enhancer binding protein β (C/EBPβ) and peroxi‐some proliferator‐activated receptor γ (PPARγ)] to the housekeeping gene GAPDH in each sample. The relative gene expression values were evaluated by the 2^‐△△Ct^ method. Sense and antisense primers were listed as follows: GAPDH: 5′‐GCAAGTTCAACGGCACAG‐3′, 5′‐CGCCAGTAGACTCCACGAC‐3′; TAZ: 5′‐GTCACCAACAGTAGCTCAGATC‐3′, 5′‐AGTGATTACAGCCAGGTTAGAAAG‐3′; RUNX2: 5′‐GGACTGGGTATGGTTTGTAT‐3′, 5′‐GCTGAAGAGGCTGTTTGA‐3′; OCN: 5′‐ACCACATCGGCTTTCAGG‐3′, 5′‐CATAGGGCTGGGAGGTCA‐3′; C/EBPβ: 5′‐GCGGGGTTGTTGATGTTT‐3′, 5′‐CTTTAATGCTCGAAACGG‐3′; PPARγ: 5′‐CCTTGCTGTGGGGATGTCTCA‐3′, 5′‐CTCCTTCTCGGCCTGTGGCAT‐3′.

### 2.9 Western blotting analysis

Cells were seeded in 60‐mm plastic dishes (Costar) for total protein isolation. Proteins were separated by 12% sodium dodecyl sulfate polyacrylamide gel electrophoresis (SDS‐PAGE) and transferred to a polyvinylidene fluoride membrane using a semidry transfer apparatus (Hoefer) for 1.5 hours at room temperature. Membranes were blocked with 5% milk in Tris‐buffered saline mixed with tween20 (TBST) for 2 hours at 37°C, and incubated with primary antibodies against TAZ (1:1000, Cell Signalling, USA), RUNX2 (1:200, Boster, China), OCN (1:200, Boster, China), C/EBPβ (1:200, Boster, China), PPARγ (1:200, Boster, China), or GAPDH (1:3000, Bioworld, USA) at 4°C overnight. Then the membranes incubated with IRDye800^®^ conjugated secondary antibody (1:20,000, Rockland, USA) for 1 hour at 37°C, following scanning with the Odyssey Infrared Imaging System (Li‐COR Biosciences). Then the integrated intensity for each detected band was determined with Image J, v.1.46.

### 2.10 Inhibitor study

Ten micromoles per litre MEK‐ERK inhibitor U0126 (Beyotime Institute of Biotechnology, China) and PI3K‐Akt inhibitor LY294002 (Beyotime Institute of Biotechnology, China) were added simultaneously into differential medium after 24 hours’ cell attachment. Then the expression of TAZ was detected by real‐time RT‐PCR and Western blotting analyses.

### 2.11 Statistics

Quantitative results were expressed as mean ± standard deviation (SD). All experiments were replicated at least three times. Kolmogorov‐Smirnov test was used to verify the normal distribution of variables. Independent samples *t* test for comparison of two groups, one way analysis of variance (ANOVA) followed by Student Newman Keuls (S–N–K) post hoc analysis for parametric data among multiple groups, and Kruskal‐Wallis test for non‐parametric data among multiple groups were performed using SPSS, v.20.0. Values were considered statistically significant at *P* < 0.05.

## 3 RESULTS

### 3.1 Sal B had no obvious toxicity effects on cell proliferation during differentiation

**Figure 1:**
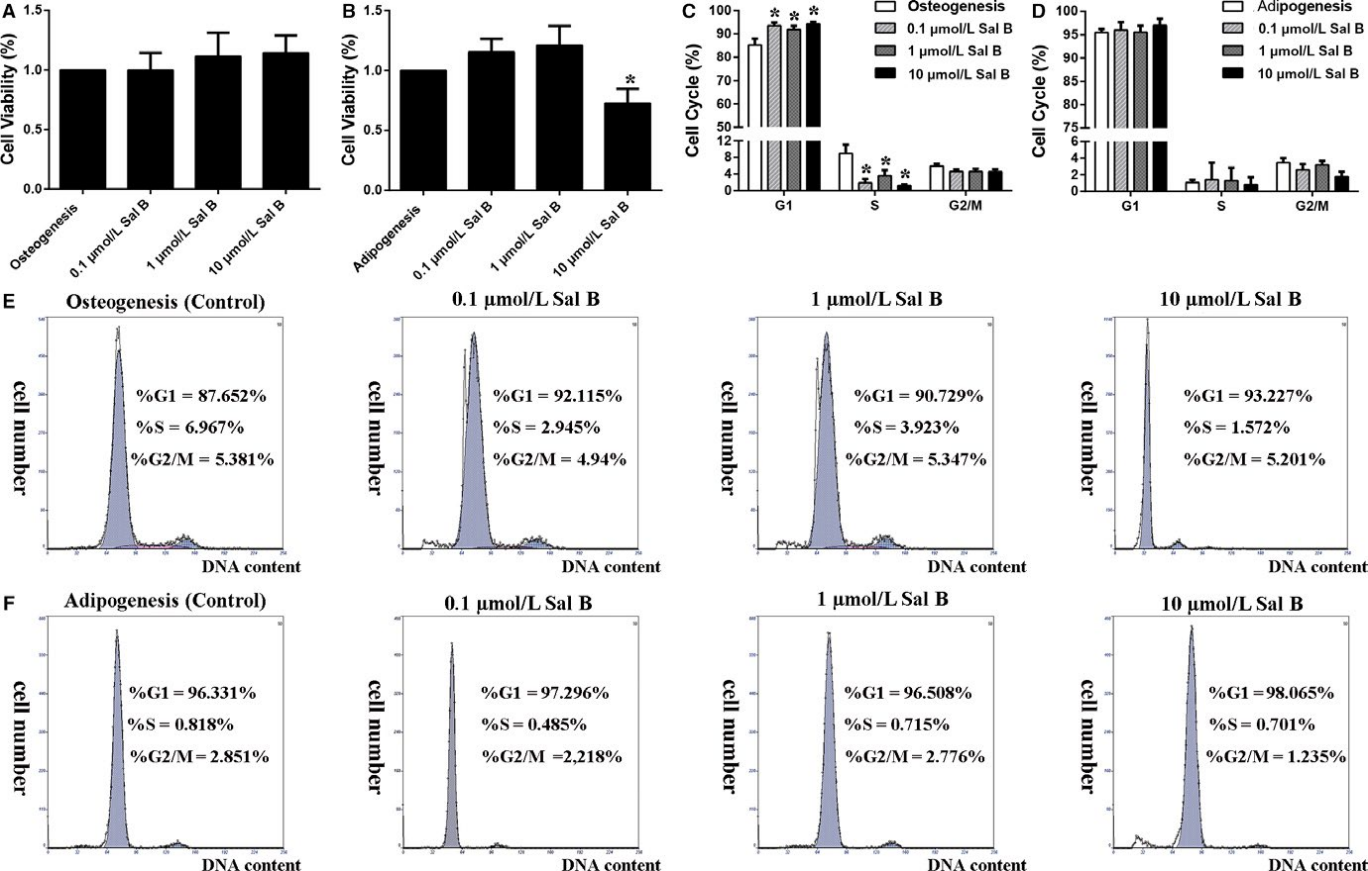
Sal B at different concentrations had no obvious toxicity effects on cell proliferation during osteogenesis and adipogenesis. (A) Cell viability was measured after 3 days’ introduction of Sal B at different concentrations (0.1 μmol/L, 1 μmol/L and 10 μmol/L Sal B) with osteogenic medium or (B) adipogenic cocktail. (C) Flow cytometry experiments were performed during osteogenic differentiation or (D) adipogenic differentiation. (E) The representative percentages of cells in G1, S and G2 phases of the cell cycles were shown in each individual graph of the MC3T3‐E1 cells or (F) of the 3T3‐L1 cells. Bar graphs showed the means ± SD from at least three independent experiments. (n ≥ 3) *P < 0.05 vs the control group (cells cultured in osteogenic or adipogenic medium without Sal B)

Initially, we tested whether or not Sal B at different concentrations (0.1 μmol/L, 1 μmol/L and 10 μmol/L Sal B) affected the cell proliferation during osteogenesis and adipogenesis. Preliminarily, the MTT assays showed that the cell viability of all Sal B treated cells had no differences during osteogenesis of MC3T3‐E1 cells (Figure 1A). Of interest, the cell viability of 3T3‐L1 cells was decreased after 10 μmol/L Sal B administration, while 1μM and 0.1 μmol/L Sal B had no effects on the cell viability during adipogenesis (Figure 1B). Similar to the MTT analysis, the cell cycles measured by the flow cytometer indicated that Sal B at different concentrations has no effects on the cell cycles of 3T3‐L1 cells during adipogenic differentiation. In all Sal B treated MC3T3‐E1 cells, the percentage of cells in G1 phase was significantly increased compared to untreated cells. Although any of the three concentrations of Sal B might block the cell cycle in G1 moving to S phase, the cell percentage in G2/M phase has no differences among the Sal B administration groups and the control group (Figure 1C‐F). Nonetheless, there were no obvious toxicity effects of 1 μmol/L and 0.1 μmol/L Sal B administration on the proliferation of the MC3T3‐E1 or 3T3‐L1 cells, respectively.

### 3.2 Sal B facilitated osteogenic differentiation and increased the TAZ expression

**Figure 2:**
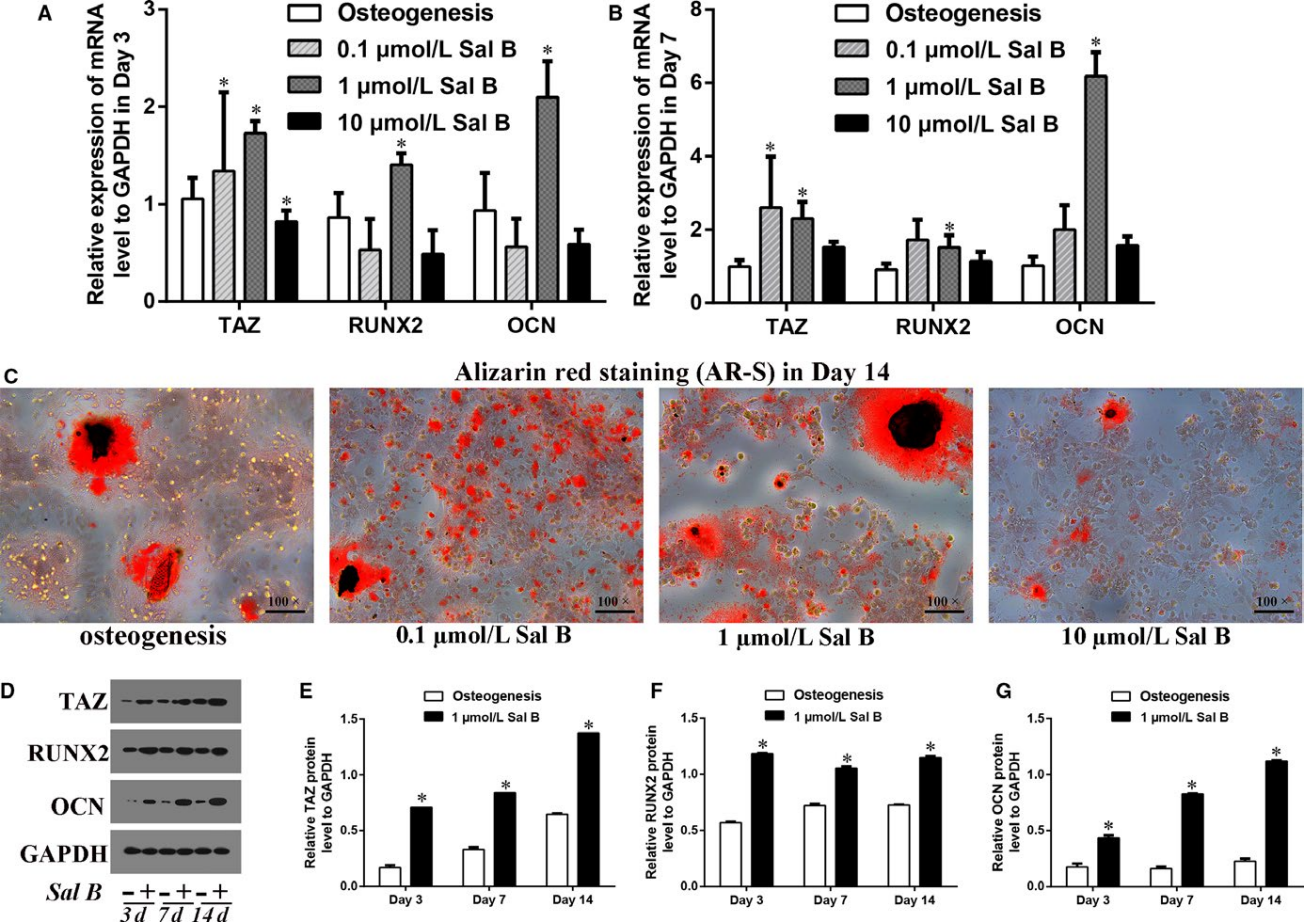
Sal B facilitated osteogenic differentiation and increased the TAZ expression in MC3T3‐E1 cells. (A) The relative expression of the TAZ, RUNX2 and OCN mRNA levels to GAPDH were presented in Day 3 and (B) in Day 7 after the induction of osteogenic medium in the absence or presence of Sal B at different concentrations (0.1 μmol/L, 1 μmol/L and 10 μmol/L Sal B). (C) The alizarin red staining (AR‐S) reflected that the calcium droplets in Day 14 after the treatment. (D) The relative protein levels of TAZ, RUNX2 and OCN to GAPDH were presented in Day 3, in Day 7 and in Day 14 after the treatment. (E‐G) Bar graphs showed the means ± SD of the relative protein levels of TAZ, RUNX2 and OCN to GAPDH from 3 independent experiments. (n = 3) *P < 0.05 vs the control group (cells cultured in osteogenic medium without Sal B)

Further to verify the effects of Sal B on cell differentiation, MC3T3‐E1 were treated with Sal B at three different concentrations. Real‐time RT‐PCR analysis showed that the higher expression of TAZ, RUNX2 and OCN appeared in 1 μmol/L Sal B administration group in Day 3 as well as in Day 7 after the induction of osteogenic medium (Figure 2A,B). Also, the AR‐S indicated that both 0.1 μmol/L and 1 μmol/L Sal B facilitated calcium deposition during osteoblatogenesis in Day 14 (Figure 2C). Specifically, the relative protein levels of TAZ, RUNX2 and OCN to GAPDH were markedly increased by 1 μmol/L Sal B compared with their expression in osteogenic cells cultured without Sal B (Figure 2D‐G). Thus, Sal B might facilitate osteogenic differentiation and increased the TAZ expression during osteoblastogenesis of MC3T3‐E1 cells, with a peak at 1 μmol/L. Accordingly, we used Sal B at the concentration of 1 μmol/L to induce osteogenesis in subsequent experiments.

### 3.3 Sal B inhibited adipogenic differentiation by increasing TAZ expression

**Figure 3:**
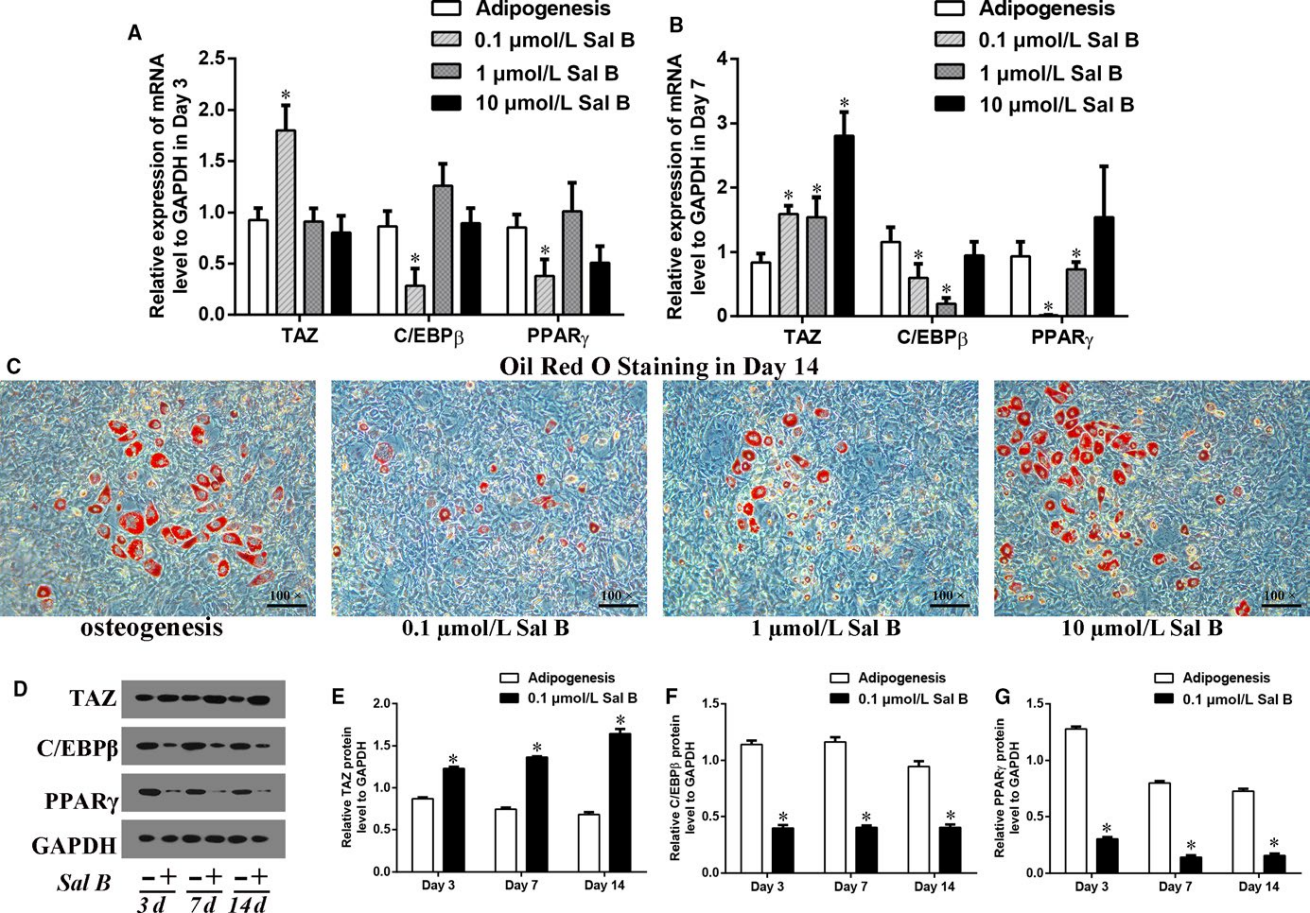
0.1 μmol/L Sal B inhibited adipogenic differentiation by increasing TAZ expression in 3T3‐L1 cells. (A) The relative expression of the TAZ, C/EBPβ and PPARγ mRNA levels to GAPDH were presented in Day 3 and (B) in Day 7 after the induction of osteogenic medium in the absence or presence of Sal B at different concentrations (0.1 μmol/L, 1 μmol/L and 10 μmol/L Sal B). (C) The oil red o staining reflected the lipid droplets in Day 14 after the treatment. (D) The relative protein levels of TAZ, C/EBPβ and PPARγ were presented in Day 3, in Day 7 and in Day 14 after the treatment. (E‐G) Bar graphs showed the means ± SD of the relative protein levels of TAZ, C/EBPβ and PPARγ to GAPDH from three independent experiments. (n = 3) *P < 0.05 vs the control group (cells cultured in adipogenic medium without Sal B)

As for the effects of Sal B on adipogenesis, we used 3T3‐L1 cell line as a pre‐adipogenic cells model and treated the cells with Sal B at the three different concentrationsl. Real‐time RT‐PCR results suggested that a higher TAZ expression and the lower C/EBPβ and PPARγ expression simultaneously appeared in 0.1 μmol/L Sal B administration group compared to the control group in Day 3 and in Day 7 after the induction of adipogenic cocktail (Figure 3A,B). Also, the oil red o staining indicated that both 0.1 μmol/L and 1 μmol/L Sal B decreased the lipid droplets after 14 days’ adipogenesis (Figure 3C). Specifically, the relative TAZ protein levels were increased and the relative C/EBPβ and PPARγ protein levels were markedly decreased by 0.1μM Sal B compared with their expression in adipogenic cells cultured without Sal B (Figure 3D‐G). These results indicated that 0.1 μmol/L Sal B might inhibit adipogenic differentiation by increasing TAZ expression during adipocytes maturation of 3T3‐L1 cells. Then, we used Sal B at the concentration of 0.1 μmol/L during adipogenesis in subsequent experiments, which was different from the concentration of Sal B used in MC3T3‐E1 cell study.

### 3.4 TAZ knock‐down offset the Sal B induced osteogenesis

**Figure 4:**
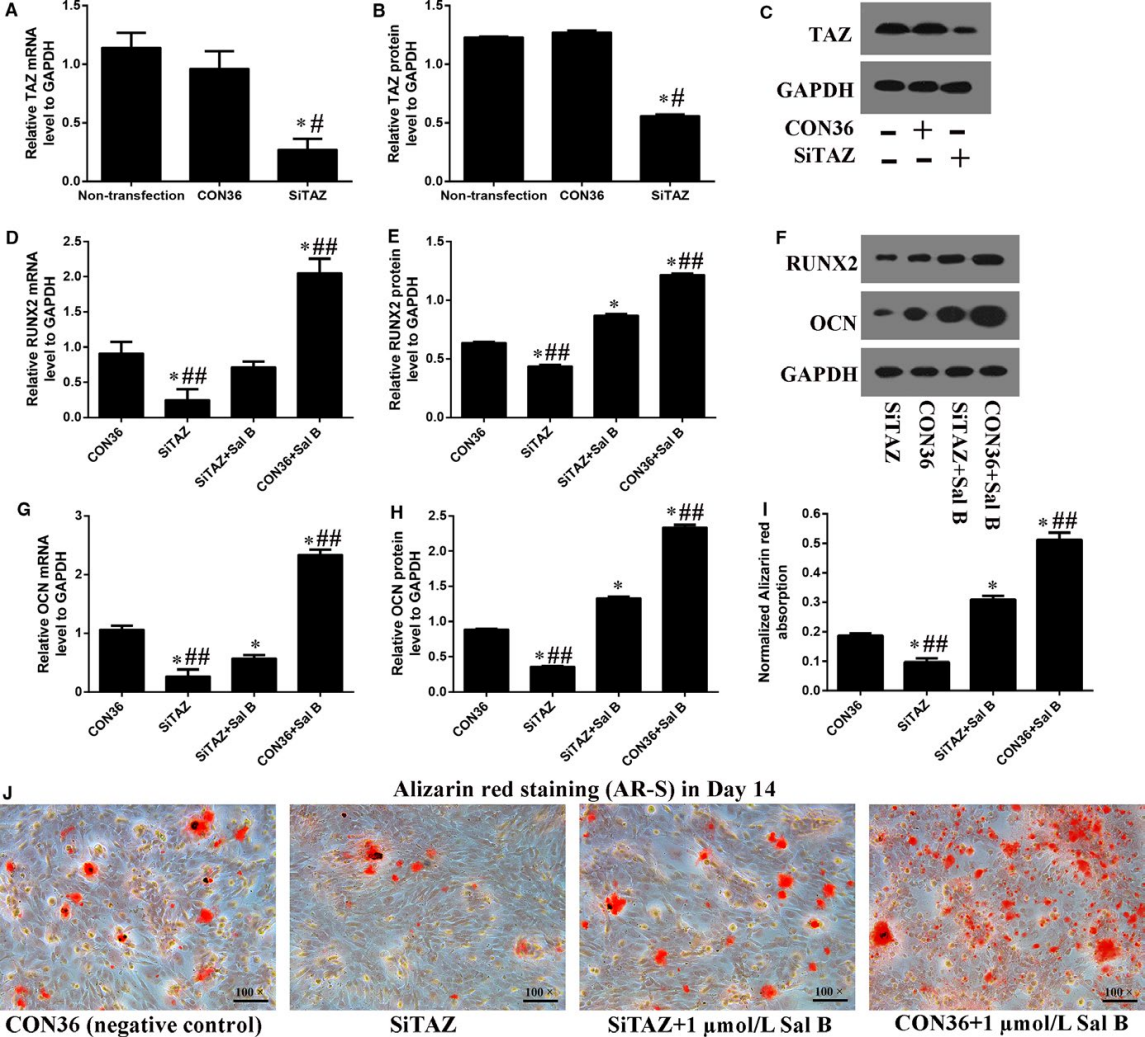
TAZ knock‐down offset the Sal B induced osteogenesis in MC3T3‐E1 cells. (A‐C) Realtime RT‐PCR and Western blotting analysis suggested that the TAZ expression were significantly knocked down by the SiTAZ plasmid compared with the negative control and non‐transfected cells. (D‐H) TAZ knock‐down offset the Sal B induced up‐expression of RUNX2 and OCN in Day 3 after the introduction of osteogenic medium. (I, J) The quantification AR‐S results showed that TAZ knock‐down reduced the Sal B induced calcium droplets. Bar graphs showed the means ± SD from three independent experiments. (n = 3) ^#^ *P* < 0.05 vs the non‐transfection group; *P < 0.05 vs CON36, the negative control group; ^##^ *P* < 0.05 vs SiTAZ + Sal B group

Transfected with plasmids containing SiTAZ sequences, the TAZ expression was knocked down in MC3T3‐E1. GFP + cells were also observed with fluorescence microscopy (Figure S2A). Transfection efficiency of the SiTAZ plasmid and its negative control plasmid (CON36) was high enough to be comparable among groups (Figure S2B). Both real‐time RT‐PCR and Western blotting analyses suggested that SiTAZ transfection significantly decreased TAZ expression compared with the negative control (CON36) and non‐transfected cells (Figure 4A‐C). Notably, TAZ knock‐down offset the Sal B induced up‐regulation of RUNX2 and OCN expression in Day 3 after the induction of osteogenic medium (Figure 4D‐H). At first, SiTAZ transfection significantly decreased the RUNX2 and OCN expression compared with the negative control (CON36). Meanwhile, Sal B administration markedly increased the RUNX2 and OCN expression. Finally, the RUNX2 and OCN expression in SiTAZ + Sal B group were lower than that in CON36 + Sal B group and higher than that in SiTAZ group. Similarly, the AR‐S results in Day 14 after the treatment were consistent with the real‐time RT‐PCR and Western blotting analyses, and verified the discount of the Sal B facilitated osteogenic differentiation by SiTAZ (Figure 4I,J).

### 3.5 TAZ knock‐down attenuated the Sal B inhibited adipogenesis

**Figure 5:**
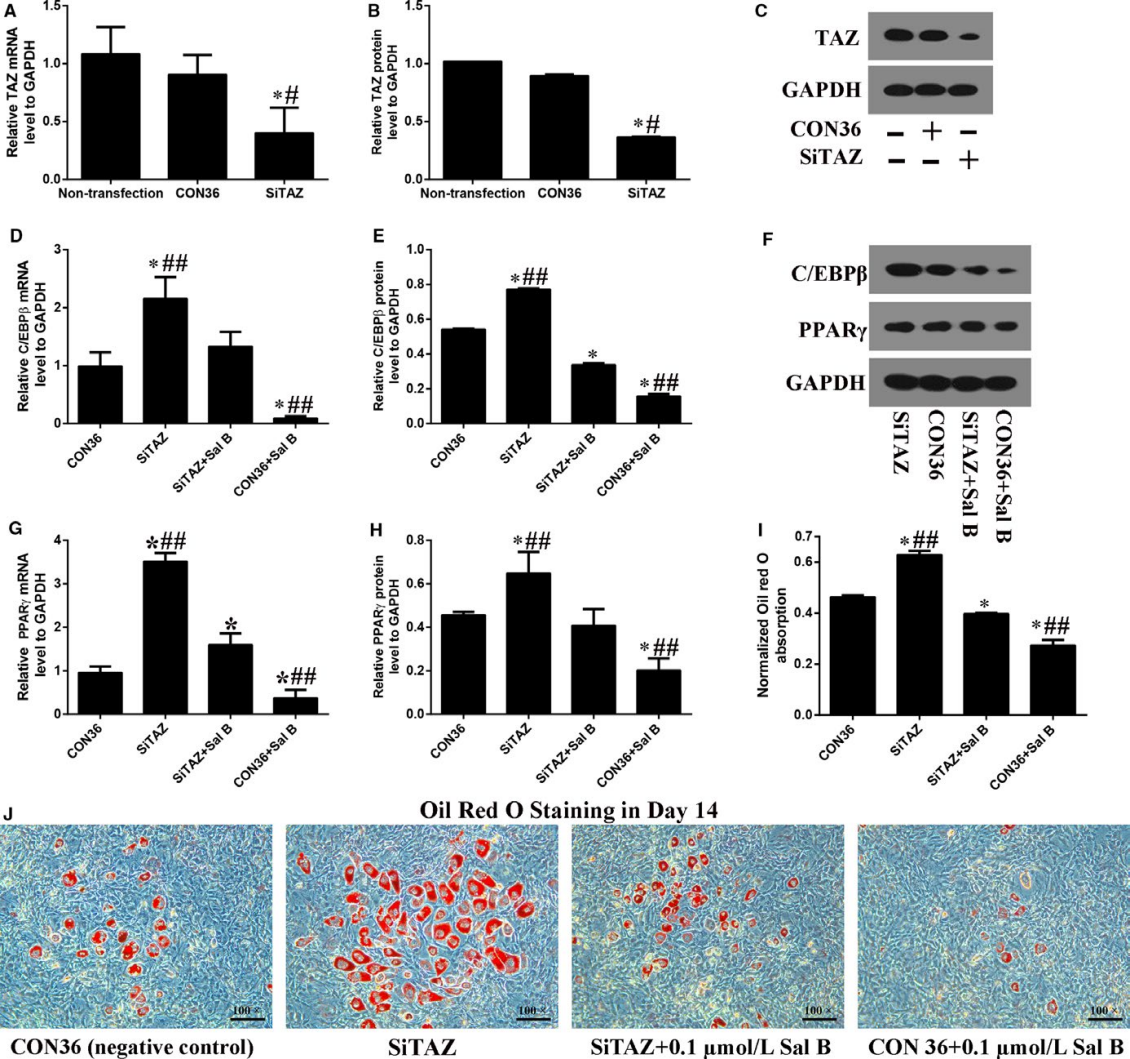
TAZ knock‐down attenuated the Sal B inhibited adipogenesis in 3T3‐L1 cells. (A‐C) Real‐time RT‐PCR and Western blotting analysis suggested that the TAZ expression were significantly knocked down by the SiTAZ plasmid compared with the negative control and non‐transfected cells. (D‐H) TAZ knock‐down offset the Sal B decreased expression of C/EBPβ and PPARγ in Day 3 after the introduction of adipogenic medium. (I, J) The oil red o staining results showed that TAZ knock‐down attenuated the Sal B decreased lipid droplets. Bar graphs showed the means ± SD from three independent experiments. (n = 3) ^#^ *P* < 0.05 vs the non‐transfection group; *P < 0.05 vs CON36, the negative control group; ^##^ *P* < 0.05 vs SiTAZ + Sal B group

In order to confirm that TAZ was involeved in Sal B inhibited adipogenesis, we then knocked down the TAZ expression in 3T3‐L1 as well. Both real‐time RT‐PCR and Western blotting analyses suggested that the TAZ expression were significantly decreased by SiTAZ plasmid transfection in 3T3‐L1 cells (Figure 5A‐C). Further, TAZ knock‐down attenuated the Sal B induced down‐regulation of the C/EBPβ and PPARγ expression in Day 3 after the induction of adipogenic cocktail (Figure 5D‐H). Initially, SiTAZ transfection significantly induced the up‐expression of C/EBPβ and PPARγ compared with the negative control (CON36). Then, Sal B administration markedly decreased the C/EBPβ and PPARγ expression. As a result, the C/EBPβ and PPARγ expression in SiTAZ + Sal B group were higher than that in CON36 + Sal B group and lower than that in SiTAZ group. Similarly, the oil red o staining quantification were consistent with the above results and suggested that TAZ knock‐down attenuated the Sal B decreased lipid droplets in Day 14 after the treatment (Figure 5I,J).

### 3.6 Sal B increased p‐ERK/ERK ratio to regulate the TAZ expression and cell differentiation

**Figure 6:**
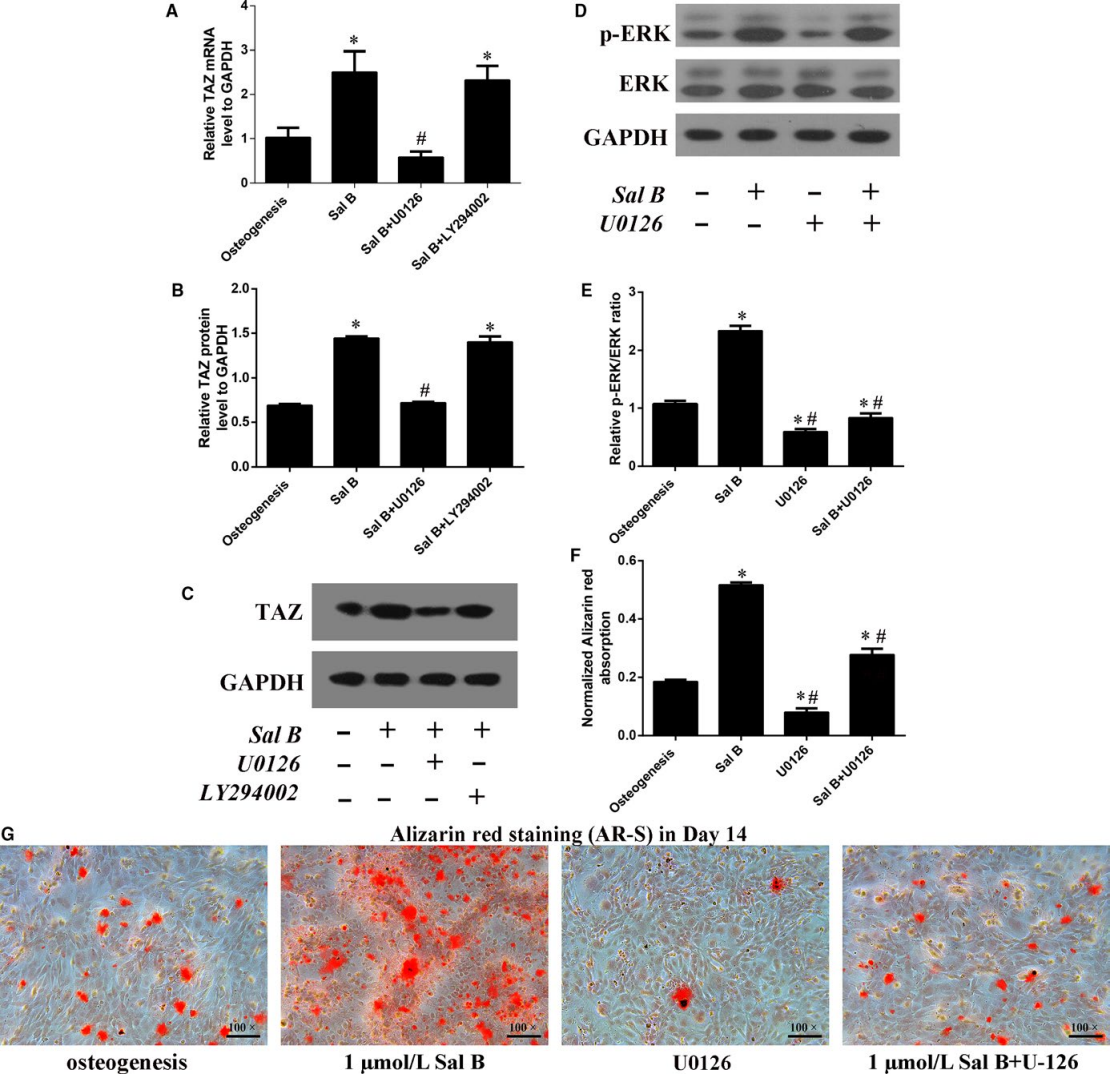
Sal B induced TAZ up‐expression was mediated by the MEK‐ERK signalling pathway during osteogenic differentiation in MC3T3‐E1 cells. (A) Real‐time RT‐PCR and (B) Western blotting analysis for the relative TAZ expression in Day 3 after the treatment. (C) Bar graphs showed the means ± SD from three independent experiments of the relative TAZ protein levels to GAPDH. (D, E) Western blotting showed that Sal B significantly lifted the p‐ERK/ERK ratio and U0126 blocked this regulation in Day 3 after the treatment. (F, G) The AR‐S quantification confirmed the involvement of MEK‐ERK signalling pathway in the facilitation of the osteogenic differentiation induced by Sal B in Day 14. (n = 3) *P < 0.05 vs the control group (cells cultured in osteogenic medium); ^#^ *P* < 0.05 vs the Sal B administration group

**Figure 7:**
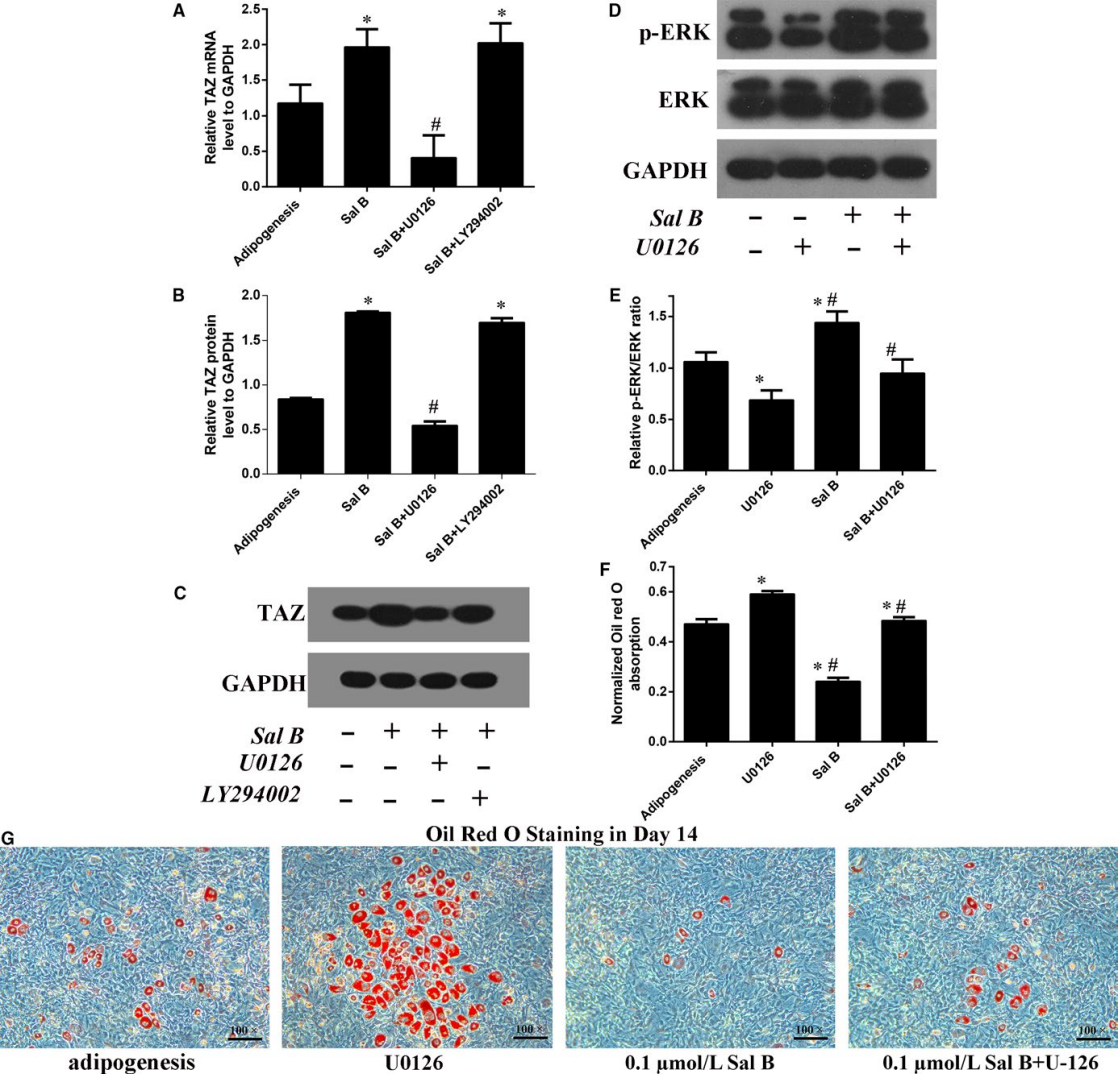
Sal B induced TAZ up‐expression was mediated by the MEK‐ERK signalling pathway during adipogenic differentiation in 3T3‐L1 cells. (A) Real‐time RT‐PCR and (B) Western blotting analysis for the relative TAZ expression in Day 3 after the treatment. (C) Bar graphs showed the means ± SD from three independent experiments of the relative TAZ protein levels to GAPDH. (D, E) Western blotting showed that Sal B significantly lifted the p‐ERK/ERK ratio and U0126 could blocked this regulation in Day 3 after the treatment. (F, G) The oil red o staining quantification confirmed the involvement of MEK‐ERK signalling pathway in the inhibition of the adipogenic differentiation induced by Sal B in Day 14. (n = 3) *P < 0.05 vs the control group (cells cultured in adipogenic medium); ^#^ *P* < 0.05 vs the Sal B administration group

UO126 and LY294002 are the inhibitors for the MEK‐ERK pathway and the PI3K‐Akt pathway, respectively. The real‐time RT‐PCR and Western blotting analyses showed that the TAZ expression were significantly higher in Sal B treatment group than that in the control group (the osteogenic group) in Day 3 after the treatment (Figure 6A‐C). Then, the up‐expression of TAZ induced by Sal B administration was significantly reduced by U0126. However, there was no significant difference in TAZ expression between Sal B + LY294002 and Sal B treatment group (Figure 6A,B). Moreover, the Western blotting analysis in Day 3 after the treatment revealed that Sal B increased the p‐ERK/ERK ratio to mediate the activation of MEK‐ERK signalling pathway during osteoblastogenesis (Figure 6D,E). Additionally, the AR‐S quantification also confirmed that U0126 offset the facilitation of the calcium droplets induced by Sal B after 14 days’ osteogenesis (Figure 6F,G). In 3T3‐L1 cells, the results were consistent with that in MC3T3‐E1 cells (Figure 7). In summary, we speculated that MEK‐ERK signalling pathway was involved in the regulation of the TAZ up‐expression induced by Sal B so thus to facilitate the osteogenesis at the expense of the reduced adipogenesis (Figure S3).

## 4 DISCUSSION

Bone diseases are now considered as common causes of lower life quality in aging people.^22^ Imbalance of bone remodeling under osteoporosis conditions have always been triggered by less osteogenesis accompanied by more adipogenesis.^4^ Thus, the ideal treatment for osteoporosis is to promote bone tissue formation from more osteoblast differentiation while preventing adipocytes accumulation in bone. In this study, we have confirmed that Sal B facilitates osteogenesis at the expense of the reduced adipogenesis by up‐regulation of the TAZ expression. Of course, TAZ knock‐down could offset the effects of Sal B on the cells differentiation. Furthermore, the p‐ERK were activated by Sal B administration and U0126 (the MEK‐ERK inhibitor) blocked the Sal B increased TAZ expression and discount the effects of Sal B on the cells differentiations. Taken together, Sal B might be considered as a therapeutic agent for the management of bone diseases.

Emerging evidences confirmed that many Chinese herbs played important roles in accelerating bone remodeling to promote fracture healing.^23^, ^24^ Salvia miltiorrhiza, one of the Chinese medicine, has been effectively used for treating bone diseases.^13^, ^27^, ^28^ As the most active constituent of water‐soluble salvianolic acid substances in Salvia miltiorrhiza, Sal B has been reported to be involved in the balance of bone remodeling.^14^, ^15^, ^28^, ^29^ Xu et al^29^ found that Sal B promotes osteogenesis of human MSCs through activating MEK‐ERK signalling pathway. In this research, we confirmed the anabolic effects of Sal B on osteoblasts maturation in MC3T3‐E1 cells. One micromole of Sal B administration could directly increase the expression of osteogenic markers, RUNX2 and OCN. RUNX2 is known to be a critical and early regulator of osteogenic development and OCN is often used as a late marker for bone formation.^30^, ^31^ Meanwhile, we also discovered a catabolic effects of Sal B on adipogenesis in 3T3‐L1 cells. A concentration of 0.1 μmol/L Sal B administration could directly decrease the expression of adipogenic markers, C/EBPβ and PPARγ. C/EBPβ is activated in the early phase of adipogenesis and plays positive roles in adipocytes maturation.^33^ PPARγ belongs to the nuclear receptor family and acted as the regulator of both adipogenesis and osteogenesis at transcriptional and translational levels.^30^, ^34^ As a part of differentiation, osteoblasts were observed to proliferate in a significantly elevated way.^35^ Hence, a successful bone formation enhancer would have no toxicity effects on cells proliferation. Our results also revealed that 0.1 μmol/L and 1 μmol/L Sal B had no obvious effects on cell viability and cell cycles. Therefore, our study proved the involvement of Sal B in osteogenic and adipogenic differentiation balance.

Previously, our team have revealed that insulin‐like growth factor 1 (IGF‐1) and GLP‐1 receptor agonist (GLP‐1RA) promoted osteoblastogenesis by increasing TAZ expression.^21^, ^36^ In this study, we defined a new signalling transduction concerning TAZ whereby Sal B regulated the switch between osteogenesis and adipogenesis. Firstly, Sal B administration up‐regulated the TAZ expression at mRNA and protein levels during both osteogenic and adipogenic differentiation. Secondly, knocking down TAZ could offset the effects of Sal B on the expression of osteogenic markers and adipogenic markers. Additionally, the quantification staining (AR‐S during osteogenesis and oil red o staining during adipogenesis) results supported our speculation. TAZ, a transcriptional modulator, could interact with kinds of transcription factors to influence the stem cells fate determination.^37^ As reported, TAZ binds strongly to the Pro‐Pro‐ X‐Tyr motif found within regulatory regions of RUNX2 and PPARγ.^10^, ^21^, ^38^ It interacted with RUNX2 and co‐activates RUNX2‐dependent gene transcription, while interacting with PPARγ and repressing its downstream target gene expression.^38^, ^39^ Consistently, our results suggested that Sal B at least partially targeted TAZ to regulate the cells differentiation into osteoblasts or adipocytes.

Of interest, our study also pointed a molecular link of MEK‐ERK pathway to the TAZ‐related switch between osteoblast and adipocyte differentiation after Sal B administration. Note that Sal B lifted p‐ERK to activate the MEK‐ERK pathway and increase TAZ expression. The MEK‐ERK signalling pathway has been intensively investigated in regulating cells differentiation.^30^, ^42^, ^43^ Jaiswal et al have suggested that the commitment of human MSCs into osteogenic or adipogenic lineages was governed by activation or inhibition of ERK1/2, respectively.^29^, ^42^ The fibroblast growth factor 2 (FGF2) was also reported to stimulate osteogenic differentiation through MEK‐ERK induced TAZ expression by Korea researchers.^45^ Previously, our team has revealed that IGF‐1 targeted TAZ by activating the MEK‐ERK signalling pathway in rat MSCs.^20^ In this study, this regulation network between MEK‐ERK and TAZ was acted as well during cells differentiation after Sal B administration, indicating a positive interaction of MEK‐ERK with TAZ.

With the progressive aging in general population, bone loss becomes a growing public problem.^46^ Pressing requirements for the treatment of bone repair is to identify anabolic agents that can increase bone formation as well as decrease fat accumulation. This study confirms the positive role of Sal B in the facilitation of osteogenesis at the expense of the reduced adipogenesis by increasing TAZ expression, pointed a molecular link of MEK‐ERK pathway to the TAZ‐related switch of cells differentiation after Sal B administration, and provided evidence to use Sal B as a potential therapeutic agent for the management of bone repair clinically.

## CONFLICT OF INTERESTS

The authors confirm that there is no conflict of interests.

## AUTHOR CONTRIBUTIONS

Peng Xue and Na Wang designed the research; Chang Liu, and Ziyi Li performed the research; Peng Xue analysed the data; Na Wang and Yukun Li wrote the manuscript.
