# Supplementary figures and images for: Sal B targets TAZ to facilitate osteogenesis and reduce adipogenesis through MEK‐ERK pathway
Source: J Cell Mol Med. 2019 Mar 25;23(5):3683–95. doi: 10.1111/jcmm.14272 (PMC6484321; doi:10.1111/jcmm.14272)

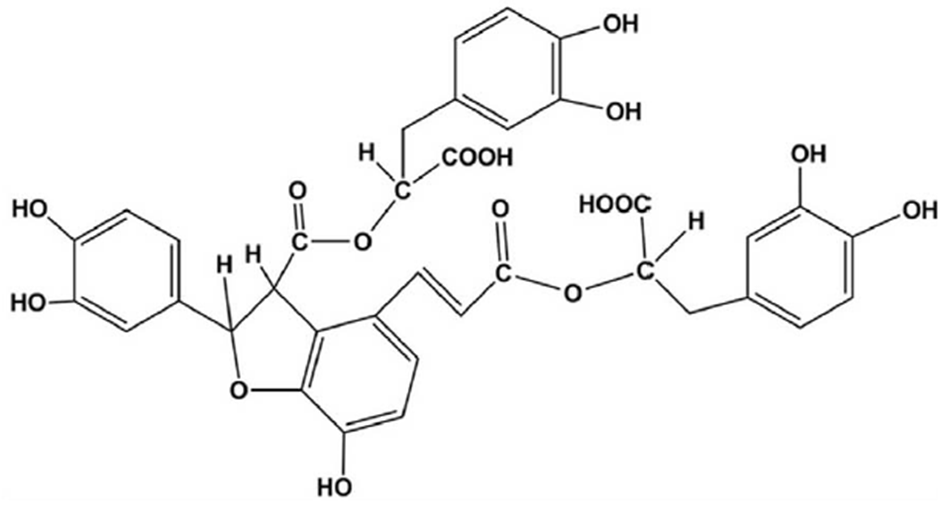

Supplement: Supplementary file 1 [file JCMM-23-3683-s001.tif]

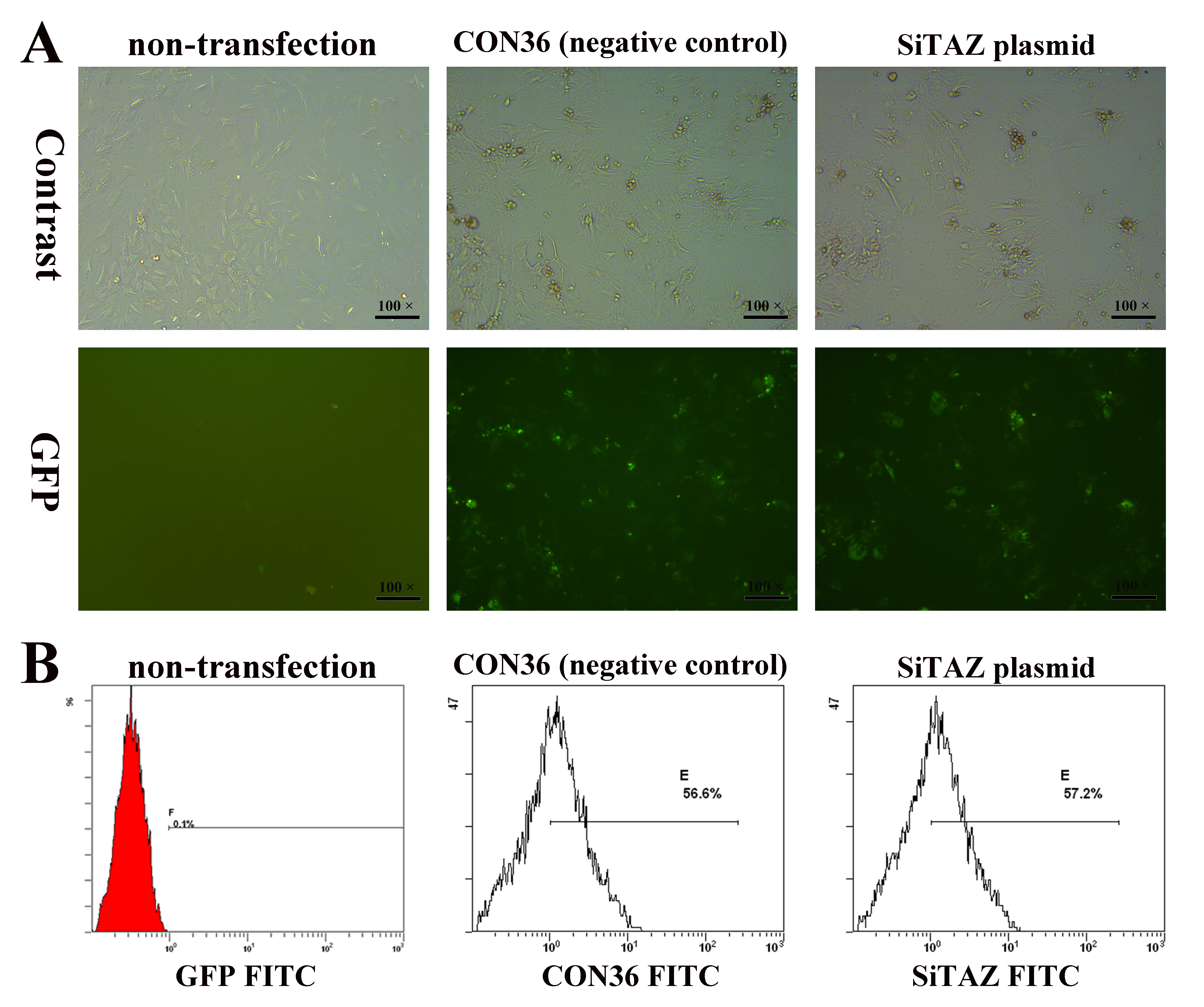

Supplement: Supplementary file 2 [file JCMM-23-3683-s002.jpg]

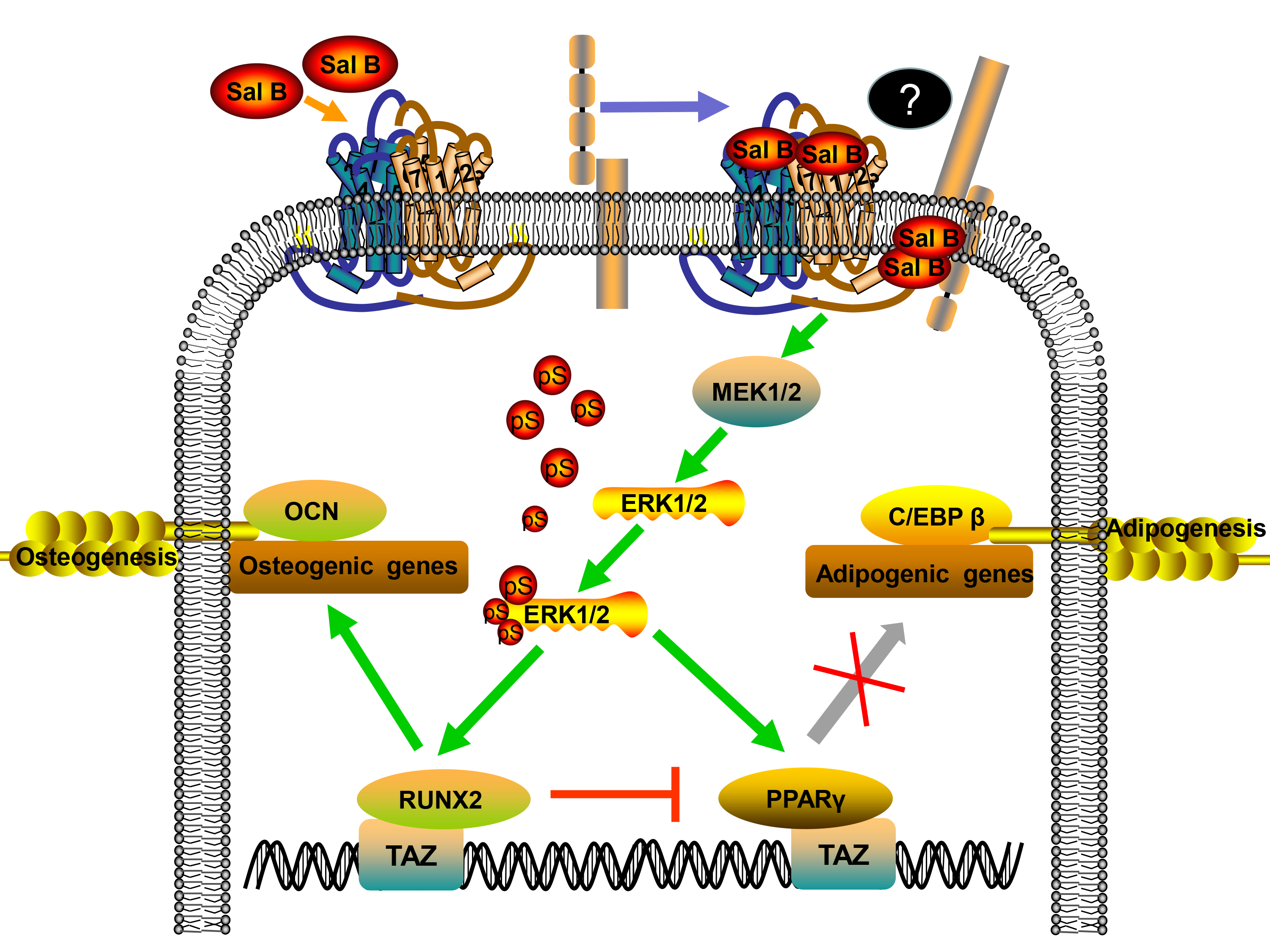

Supplement: Supplementary file 3 [file JCMM-23-3683-s003.tif]
